# Anatomical, Pathological, and Therapeutic Researches on the Yellow Fever of Gibraltar of 1828

**Published:** 1840-10

**Authors:** 


### Art. XI.

*Anatomical, Pathological, and Therapeutic Researches on the Yellow Fever of Gibraltar of* 1828. By P. C. H. A. Louis, Physician to the Hôtel Dieu, &c.; *from observations taken by himself and M. Trousseau, as Members of the French Commission at Gibraltar.* Translated from the Manuscript by G. C. Shattuck, m.d., Member of the Society for Medical Observation at Paris, &c.—*Boston, United States*, 1839. 8vo, pp. 374.

These "Researches" run the risk of being neglected by ordinary readers, as being destitute of the charm of novelty, for they refer to an epidemic which prevailed twelve years ago, and, though at that time drawing upon it the attention of all Europe and of this country more especially, is now almost forgotten but by a few medical *literati* and officers in the public service. Should it prove, however, (and we feel very confident it will prove,) that what is pathologically true of the epidemic of Gibraltar is equally true of every epidemic of yellow fever, then should this volume be regarded not as a treatise on a by-gone disease, but as a contribution, and (proceeding from the pen of Louis) an important contribution to our *general* knowledge of one of the most direful scourges of the human family, and one in which this country above all others, the United States scarcely excepted, is most materially interested. Should our supposition prove correct, that the facts observed at Gibraltar are general facts, true of yellow fever wheresoever it prevails, and the intelligent translator of this volume has already produced evidence from Martinique and from Boston (U. S.) confirmatory of this view, it tends materially to obviate an objection frequently urged against the utility of the method of observation inculcated by Louis, the speciality and peculiarity of every individual case of disease setting at defiance all endeavours at generalization, in short, the application of the Baconian philosophy to medicine. That the phenomena of disease are less invariable and fixed than those of inanimate nature, or even than many physiological phenomena, and that the close application of the inductive philosophy to the former class is beset with many difficulties, we willingly acknowledge; but for this very reason would we regard the efforts of M. Louis to surmount these difficulties as worthy of especial praise; and as far as our power extends we shall continue to encourage these and similar efforts, having a conviction that only by their success can medicine become a science, be rescued from the ever-fleeting speculations which have disgraced its annals from the days of Galen to those of Broussais.

M. Louis's estimate of his own work is moderate, for he regards it as a treatise on the Gibraltar epidemic only, not as one on yellow fever generally. This question may ultimately be decided against our author. But however this may be, it is material that the reader should know that the circumstances under which these investigations were pursued were of a nature to secure the authenticity of the facts detailed. A commission was appointed by the French government to investigate the epidemic yellow fever which prevailed in Gibraltar in 1828. This commission, consisting of MM. Gendrin, Trousseau, and Louis, reached the garrison on the 23d of November, thirty-three days before the termination of the epidemic.

"The commission commenced their labours the day after that on which they arrived at Gibraltar; the autopsies were nearly all made in the presence of the three physicians, and M. Trousseau and myself held alternately the pen and the scalpel. The symptoms of the disease were noted by him or by myself, rarely by both of us, and several of our professional brethren assisted us in this part of our labours, of whom I mention particularly Mr. Fraser, surgeon of the Civil Hospital, and Messrs. Gilkrest and Smith, surgeons of the English forces, who kindly consented to be our interpreters. M. Chervin also sometimes acted for us in this capacity. In the midst of universal desolation, our observations were taken with great care. We had time enough, and our professional brethren afforded us every facility for a thorough examination of the bodies, being themselves present at the autopsies; and we, that is, M. Trousseau and myself, were fully aware of the importance of a study of the pathology of the disease, even supposing the necessary information on the origin and mode of propagation of the epidemic to be obtained in the documents collected by the commission. We felt that independently of the task which our government had imposed on us, we owed it to our profession to study the disease before us, and this, too, more carefully if not more minutely than we should have studied an ordinary malady." (Author's Introduction, p. xviii.)

In the first part of the work we have five cases detailed with so much minuteness as to occupy fifty pages. The object of this seems to be merely to prove that the disease was yellow fever, a matter on which there is not much likelihood of any controversy arising. The second part, one of great interest, contains a description founded on twenty-five autopsies, twenty-three of which are of subjects who died of yellow fever, of the external condition of the body, and the state of the viscera contained in the three great splanchnic cavities, commencing with those which deviated most rarely from their natural state. The proportionate frequency of the lesions and the period at which they were developed are pointed out. The author endeavours to estimate their relative frequency, and draws a comparison between them and what is analogous to them in subjects who die of the acute diseases of Paris. We shall present our readers with a condensed view of this very important part of the work.

*Exterior of the body.* Externally there was observable great cadaveric, rigidity, and in some the muscular prominences were as well marked as they could have been during life when the muscles were in a state of strong contraction. The skin was yellow in all except three, and when the yellowness was not well marked it was more so on the trunk and head than on the limbs. The muscles had their natural colour, firmness, and cohesion. In one case the superficial muscles of the calves and hams were infiltrated with blood. That the disposition to hemorrhage was not great is shown by the fact that in one only of twenty-three cases was there a slight exhalation of blood in the subcutaneous cellular tissue and in the superficial muscles.

*Brain.* In the arachnoid cavity, there were in four individuals about five small spoonfuls of serum, bloody in two cases, clear and limpid in other two. The infiltration of the sub-arachnoid tissue was more frequent, existing in two thirds of the cases; it was generally slight. The infiltrated fluid was limpid except in one case where it was at the same time red and abundant. In general the lateral ventricles contained some serum, generally very little. The pia mater was more or less injected in six cases. The colour of the cortical substance of the brain was altered in eight subjects, of a pale or bright red in five, violet or lilac colour in others, a colour which existed in the whole thickness and extent of the cortical substance. This substance presented no other appreciable alteration. The medullary substance had almost constantly its natural appearance, and it was found injected to any remarkable degree in four subjects only. In another it had a lilac shade in some points; in all it was of a good consistence, and there was no softening in any part of it. In the cerebellum, the cortical substance was of a rose colour in three subjects, of a violet-rose in three others; the consistence of the organ appeared generally diminished in one case only.

*The spinal marrow and its membranes.* There were found from two to four spoonfuls of clear serum in the arachnoid spinal cavity in all the cases where the spinal canal was opened. In one case the serum was more or less red. The spinal marrow itself examined in the same number of cases presented nothing remarkable.

The author in his third chapter treats of the lesions in the respiratory apparatus. The only very general morbid appearance was a livid colour, more or less marked in several cases. The laryngeal mucous membrane was of an intense red in two subjects. The same was true of the trachea in one case, and in another its mucous membrane was both red and softened, consequently inflamed. The author found the mucous membrane of the air passages in a much more natural state in this disease than in those who die of fever in Paris; but he asks the question, What disease of the kind is accompanied by such slight febrile symptoms as yellow fever?

*Lungs.* These organs were more or less smooth (?) in the greater part of the cases, but entirely natural in three subjects only.

"In five their volume was considerable; and this depended, in great part, sometimes on the dilatation of the pulmonary vesicles, sometimes on different lesions which we are about to describe. There were either black spots of from two to five lines in diameter, or masses of the same colour more or less impermeable to the air, or else they were the first and still more rarely the second degree of pneumonia. The spots were found in nine subjects, sometimes without complication, sometimes with the lesion of which we have just spoken. Usually of a brown black, rarely of a crimson hue, they were more or less concentrated, and occupied a variable space at the exterior or in the interior of the lung, and in some cases they were found only in the lower lobe. The density of the tissue, which was the seat of them, was not manifestly increased except in two cases, this increase of density being the manifest result of an effusion of blood more or less intimately combined with the pulmonary tissue. The blackish masses existed in six individuals; their consistence was greater or less, they contained no air, they had not the granulated aspect of hepatized lung, they presented but slight traces of organization, so that merely some cellular fibres irregularly disposed might be distinguished in them. Usually they could be easily broken down; in some cases also they yielded by pressure the blood of which they were almost entirely composed, and the pulmonary parenchyma remained apparently of its natural consistence." (pp. 64-6.)

M. Louis ascribes both the spots and masses to an exhalation of blood into the pulmonary parenchyma, and remarks that such are much less frequent in those who die of the acute diseases of Paris than in the victims of the yellow fever of Gibraltar; whilst on the other hand he remarks, the disposition to inflammation of the lungs is much greater in those who succumb to the latter than the former disease. The superior prevalence of effusion of blood into the pulmonary parenchyma in yellow fever should not, he says, excite surprise, considering the disposition to hemorrhage in this disease.

*Pleuræ.* These membranes were remarkable only for the absence of recent lesions of any importance.

*Organs of circulation.*

The *pericardium* was natural in all those examined, with the exception of one or two spoonfuls of yellowish serum in seven subjects.

With the exception of some deviations from the normal condition as to size, certainly not connected with yellow fever, the changes in the *heart* were in its consistence. It was flabby in seven subjects, and at least as many times its cohesion was diminished. In one case the heart is said to have been flaccid without diminution of its cohesion. In two subjects it contained no blood; a greater or less quantity of it was found in others, either liquid only or liquid and clotted; the clots were black or yellow, fibrinous. The ventricle and its lining membrane were of a bright colour in three subjects whose hearts were flaccid.

The aorta contained a greater or less quantity of clotted or liquid blood in all the subjects, and its internal surface was rosy or red in six of them, either in its whole length or, as was more commonly the case, in a part only. The redness was not continuous in all these cases. There was a communication of the colour, evidently the result of imbibition, in those who were opened late. The redness of the aorta is more frequent in those who die of acute disease in Paris than in the subjects of yellow fever.

*Digestive apparatus.*

The *pharynx* and *tonsils* presented nothing remarkable.

The *œsophagus* was devoïd of the epidermis, covering its mucous membrane, in six cases; it was partially wanting in nine others, and was perfect in five only. The mucous membrane was blackish or of a deep brown in six cases; red, colour of onion parings, or reddish brown in three others; of a proper thickness and consistence in all. The black colour was found only where the epidermis was wholly or partially destroyed. The author does not regard the destruction of the epidermis of the œsophagus as peculiar to yellow fever.

*The stomach.* There were deviations in the volume of the organ from the ordinary condition, it being above the usual size in seven and below it in three subjects; but these deviations ought not to be considered as the effect of the disease. The *contents* of this organ were not the same in all subjects. Their predominant colour was red more or less inclining to black. The duration of the disease had little appreciable influence on this colour. The red or black matter varied in quantity from four to twenty ounces, and the deeper its colour the more abundant it was. The red and black matter did not differ in consistence. The latter separated on standing into two parts: the one superior, more liquid, of a bister colour; the other inferior, less abundant, and, as it were, formed of blackish particles. It was not mixed with clots of blood; but M. Louis has no doubt of its containing it, for the vessels in which it was kept and bodies plunged in it were stained red. What was the mechanism of its formation? There was no vessel ruptured in the whole tract of the alimentary canal and no lesion of the gastric mucous membrane, so that it must be considered a product of the exhalation of this membrane. The *colour* of the mucous membrane was not the same in all cases, and it varied in the same case in different points of the surface. In one case it was universally red except near the pylorus; in five others through a more or less considerable part of its surface. Instead of a red colour there was an orange or slight rose tint, or a colour of onion parings, in a varying extent, in eight others. In two cases there was a ruddy or bister hue; a greenish or yellowish in two others. The colour of the membrane was natural in three subjects only. These changes of colour were independent of the contents of the stomach; and M. Louis is of opinion that they were not much more frequent than in those who die of the acute diseases of Paris.

The *thickness* of this membrane was natural in half the cases. In other cases it was thickened. Its *consistence* was normal in thirteen cases; in ten the membrane was softened. This softening, M. Louis thinks, was the result of inflammation in the majority of cases; in seven it was associated with thickening and redness. He remarks that "it is hardly less frequent after other acute diseases, but what makes it more important in this fever is the rapidity with which it took place. The disease was short, and the gastric mucous membrane was not affected in all the cases at the commencement of the fever, or at any rate according to all appearance it was not."

The *mamelonated appearance* was found and usually to a remarkable degree in fifteen subjects. In all the cases this appearance was associated with either thickening or softening, or both these conditions conjoined, and was evidently the product of inflammation. *Ulcerations* of the gastric mucous membrane were found in two cases only, a proportion not exceeding that observed in cases of death from other acute diseases.

The author concludes from these facts, "that the yellow fever of Gibraltar of 1828 is not a gastritis, that the different lesions of the gastric mucous membrane are secondary or accessory, and that in cases where they are found, they were probably developed at a certain period after the commencement of the disease." (p. 99.) He thinks, however, that the disease "had a particular influence on the development of this gastritis, since it was more frequent and came on nearer the commencement of the principal disease, with which, in some cases, it would appear to be confounded, than in any other acute affection." (p. 99.)

*Small intestine.* As in the case of the contents of the stomach, those of the small intestines were of various appearances. There was in one case a yellowish liquid, a more or less viscous and abundant one in five others; a reddish, brownish, blackish, or even entirely black matter in fifteen subjects, and blood recognizable by all its external characters in another. The author is of opinion that the stomach is the main source of the black matter, and that it passes thence into the intestines; but in some cases he thinks it was the product of exhalation from the mucous membrane of the intestines, since it was found there in two instances, when there was neither red nor black matter in the stomach. The general conclusion is that the presence of the black matter in the small intestine in so great a number of subjects forms an anatomical character, but a secondary one, of the yellow fever of Gibraltar, and distinguishes it from other acute diseases, in the course of which nothing of this kind is observed, or, at any rate, not oftener than twice in a thousand cases.

The *mucous membrane* itself was slightly injected or red in ten cases; it was thickened in one case only; its consistence was natural or nearly so in ten instances; it was slightly diminished in six; and in four others, through a moderate extent of the bowel, its consistence was that of mucus. This softening was certainly in most of the cases not of an inflammatory nature. Except in one case, in which some of Peyer's patches near the cæcum were slightly tumefied, there was no affection of the glands of Brunner and Peyer.

The *contents* and *condition of the great intestine* bore a considerable relation to those of the small intestine; and M. Louis remarks that excepting the presence of the black matter the lesions found in the large intestines of those who died of yellow fever were the same as those found in individuals dying of other acute diseases, and the only difference is in the relative frequency with which these lesions occur.

The *lymphatic glands* were almost always natural; those of the neck, of the mesentery, and of the biliary organs were the only ones found otherwise. M. Louis brings evidence that the swollen condition of any of these glands was not to be ascribed to inflammation of a corresponding mucous membrane. He refers their condition to the law according to which wherever febrile symptoms, whatever may be their cause, continue during a certain number of days, organs not primarily affected become the seat of alterations more or less important, usually of an inflammatory character.

*Liver*. The lesion of this organ M. Louis considers as the anatomical character of the disease which is truly diagnostic. It is true that two cases were given to the Commission as examples of yellow fever in which this diagnostic mark was wanting on examination after death. It is clearly shown, however, that these were not cases of the epidemic. This lesion consisted in a discoloration, the liver being sometimes of the colour of fresh butter, sometimes of a straw colour, sometimes of the colour of coffee and milk, sometimes of a yellowish gum colour, or finally an orange or pistachio colour. This discoloration was not the same through the whole extent of the liver; more marked in the left than the right lobe; it was also more uniform. In cases in which the colour was uniform in the left lobe there was a mixture of gum-yellow, orange, or red points, larger or smaller, in the right lobe, or else a rose tint, which did not exist in the left lobe. With this discoloration was associated a marked paleness and a diminished quantity of blood, so that wherever this appearance of the liver was decided, the sections of it were dry and of an arid appearance in the left lobe. This pale and anæmic state of the liver was unaccompanied with softening, indeed in several cases was associated with increased consistence of the organ. Though the author deems it impossible, in the present state of science, to determine the nature of this alteration, he decides, from the circumstances just enumerated and the liver being of a natural size, that it is not of inflammatory origin. He assigns sufficient reasons for the conclusion that this anæmic condition is the effect neither of hemorrhage from the intestinal canal nor of a derivation produced by the inflammation of the mucous membrane of the stomach or duodenum. He considers its commencement contemporaneous with that of the disease, or that it occurs shortly after it. It is remarkable that no other organ is in the same anæmious condition, and that many of them, as the lungs and stomach, contain a larger quantity of blood than usual. This part of the subject is concluded by M. Louis with the remark that the liver was the only organ found constantly altered in cases of death from the fever of Gibraltar; and that this alteration is not found in subjects dying of other diseases, and he consequently regards it as the anatomical character of yellow fever.

There are some anatomical matters of minor importance described in the work; but these we pass over that we may consider more at length the very valuable chapter on the causes of death in this disease. The author acknowledges at the commencement of this chapter the difficulty of the subject, a difficulty depending considerably on the obscurity of the nature of the lesion uniformly observed in the liver, and the consequent impossibility of estimating its share in the fatal event. In eight of the cases the death of the patient remained unexplained by the condition of the organs, and in five others it was *difficult* to explain it in this way. M. Louis makes some excellent remarks on the value of the former fact: he says,

"The fact is valuable on account of the consequences which flow from it. We do not observe the same in anything like such a proportion in the course of other acute diseases. For this reason it is one of the characteristics of the yellow fever, at least of that fever which prevailed in Gibraltar in 1828. And if observation shows that there is in disease something beyond what we see, that its cause has sometimes a great deal to do with the death of those who are attacked with it, this double proposition is more evident here, where we must admit that the cause of death often kills by itself, or independently of appreciable alteration of the organs, and even up to a certain point of apparent derangement of the functions. We must remember that nearly the same thing happens in many cases of poisoning. It results from the facts we are studying, that if an intimate knowledge of the lesions observed in subjects dying of yellow fever is indispensable to the practitioner, he must also remember that in this disease as in others there is something besides these lesions, and that he must profit by all that experience or chance may teach him of the best mode of treatment. The treatment called rational is less sufficient here, inasmuch as the most constant of the lesions, that which forms the anatomical character of the disease, cannot be characterized or appreciated in the present state of science, so that we cannot calculate on treating the disease successfully by any of the established modes or formulas." (pp. 145-6.)

It would unquestionably have been more gratifying had the researches of our author led to conclusions calculated to give at once more decision and success to our treatment of the disease. Such, however, it is evident from the chapter on treatment, of which we shall speedily give a view to the reader, is not now the case; and, though we are not without a hope that the researches of Louis may at a future day have an important bearing on the therapeutics of yellow fever, at present this disease is left, in common with other epidemics, to such remedies as the observation of outward and visible symptoms or even chance may suggest. We are aware that the immediate barrenness of results from the most laborious pathological investigations discourages medical men, and especially the very practical-minded medical man of this country from such pursuits. We would, however, as far as in us is, guard our professional brethren from such despondency of mind. Patience in investigation is seldom altogether unrequited, and we trust before closing this article to be enabled to show that the facts brought forth by Louis have even now an important application, though not, we admit, to the treatment of the disease. It is obvious, too, that the fact brought to light relative to the liver may ultimately prove to have a more material practical bearing than it at present possesses; it may be the initiatory but essential step to an acquaintance with the intimate nature of the disease calculated to strip it of many of its terrors.

The description of symptoms, though extremely perfect, is so long, occupying nearly 100 pages, for each individual symptom is the subject of a section, that we feel ourselves compelled to limit our extracts to the general description. In this the symptoms are described as they occurred in fatal cases; in severe cases which recovered; and in slight cases which likewise recovered. We shall present them in an abridged form, in the order observed by M. Louis.

*Fatal cases.* These commenced with an intense headach, accompanied with chills, shivering, pain in the limbs, and, soon after, pain in the back. A heat, rarely intense, succeeded to the chills, and was sometimes followed by perspiration. At the same time the countenance became red and animated, and in some cases swollen. The eyes were red, glistening, suffused, and patients often complained of a sensation of smarting in them. The thirst was intense, the anorexia complete.

Pain at the epigastrium usually came on in fifteen or twenty hours from the commencement of the disease. It was generally inconsiderable, and very few patients complained of severe or acute pain. With the epigastric pain came nausea and vomiting, excited by drinks and purgatives in several cases, spontaneous in others. The dejections were infrequent, that is, where no laxatives had been administered. The abdomen preserved its form, was supple and not painful to the touch, except in the epigastric region. The sleep was inconsiderable, some patients were restless, and in some there was a good deal of jactation during the night. The smaller number experienced as early as the third day a real anxiety, could not remain in any posture, and in some cases there was delirium; but this symptom did not usually come on till the last day of life, and for this reason is rather to be considered as belonging to the agony than the disease. Otherwise, with few exceptions, there was neither prostration nor stupor. The pulse was moderately accelerated, regular, generally bearing relation to the degree of heat, which was generally slight. The skin of the thorax was injected in some cases. This redness and that of the eyes diminished towards the middle period of the disease or a little later, and new symptoms appeared. To the injection of the integuments of the chest there succeeded a slight yellow tint of that part, and the eyes were of the same colour. When this colour appeared thirty-six or forty-eight hours before death, it became rapidly brighter, so as to be of some intensity at the time of the fatal termination. In other cases where it came on only just before death, it was slight at the autopsy, and commonly limited to the trunk. About this period or a little later the matter vomited, from being of a yellow colour, became brown or black, and the dejections blackish or black. At this period of the disease, the uncomfortable feelings and the anxiety continued during different lengths of time and in different degrees; the strength diminished, the temperature fell, so that the limbs were cold before the agony, and in a certain number of cases there was suppression of urine.

Yellow fever resembles other dangerous febrile diseases in an occasional mildness of external aspect even in fatal cases; the slightness, as M. Louis observes, of the fever and of the pains wherever seated, the absence of agitation and delirium, and the little diminution of strength impressing on the disease a character of mildness calculated to deceive at once the patients, their attendants, and the physician. It was under this form of disease that patients died without taking to their beds, on foot, as it was expressed by their friends.

The severity of the symptoms did not always correspond with that of the lesions: of these last, one was constant, the specific alteration of the liver. The inflammatory state of the mucous membrane of the stomach came next in frequency, and sometimes explained in a manner sufficiently satisfactory the symptoms that had occurred.

*Severe cases in patients who recovered.* The early symptoms differed in degree only from those in the fatal cases. In some subjects the stools became black, and in a few, and these mostly children, the brown or black vomit occurred. In a great many cases there was no yellowness, and in the majority of cases where it was found it came on from the fourth to the sixth day of the disease. The extreme restlessness, the jactation which took place in those who died was not met with in any of the cases now under consideration. Towards the fifth day, the symptoms becames less severe, the skin cooler, the pulse calm, the epigastric pain diminished or totally disappeared, the thirst was less, the appetite returned, and convalescence commenced.

*Mild cases.* These began with the usual symptoms very slight in degree. In the progress of the complaint the epigastric pains were rare, and so too were the vomitings, which were almost never spontaneous, and which in no case were of a brownish colour. So slight was the diminution of strength, that the patients either did not keep their beds at all, or were there for half a day only, thus, to use their own expression, going through the disease on foot. In several of these cases the febrile symptoms were very slight, continuing only during twenty-four or thirty-six hours; yet these persons were exempt from any other disease in the course of the epidemic, though exposed to all the causes which could have produced in them the yellow fever, and it was likewise remarked that persons who had been thus slightly affected in the epidemic of 1804 passed uninjured through the epidemics of 1818, 1824, and 1828. This mild form was principally observed in children.

Leaving the estimate of the value of individual symptoms,—though we admit that we do so with regret, for no portion of the work displays M. Louis more favorably as an admirable observer and a truly philosophic physician,—we pass to his account of the mortality of the disease. The discrepancy in the mortality of the endemic derived from two sources is really appalling, being calculated to shake our confidence in all medical testimony. According to calculation made by the Commission, from 600 individuals, short histories of whose cases had been taken, the mortality was in the proportion of one to six and a half, whilst, according to the tables prepared from the bulletins of the public authorities, published daily in the Gibraltar Gazette, giving an account of all the patients of the city and of the hospitals, the mortality was much more considerable, being 1183 out of 5383, or about as one to four and a half. These latter documents, it would appear however, were much less accurate in reality than in appearance; for, though regarding the number of the dead there could be no doubt, as they all passed through the land gate and were counted, there was not the same evidence regarding the number of sick, as many had the fever who concealed it from dread of being sent to the hospital or lazaretto. This seems to us a very probable explanation of the discrepancy, for we have known the same source of inaccuracy affecting returns in epidemics, especially in cholera, in this country, where the same motive for concealment did not exist. The mortality at Gibraltar was found to vary according to age and sex. The ratio is thus stated by the commission from the documents they collected:—of children attacked a seventh part only died, of women, one in five and a half, and of men, one in four and a half. M. Louis remarks that the same symptoms have not the same value for prognosis at all periods of life; thus the black vomit, which in men was the most certain harbinger of death, took place in a great many children who recovered.

In the third chapter, the question, "Was the disease the same at the commencement, and at the termination of the epidemic?" is considered, and with the same diligence in the collection of particulars and philosophical spirit in deriving inferences from them which are the general characteristics of the work. The author first compares the symptoms observed by the medical officers of Gibraltar during the months of September and October, and those seen by the commission from the 24th of November to the 24th of December, and finds the closest correspondence between them. He gives an example of the pathological lesions found in a case fatal on the 26th of November, and one of the last fatal cases of the epidemic examined on the 25th of December, and here, too, finds the disease preserving its identity. He subsequently shows the proportion of severe to mild cases to have been nearly the same throughout the epidemic: regarding the proportionate mortality in a given number of cases there is a considerable discrepancy between the voluminous documents furnished by the public authorities and the deductions from the comparatively scanty materials collected by the commission. This M. Louis explains by the small number of cases with which the latter body dealt, and the consequently great influence on the *ratio* of a trifling error. It is likewise found, that whilst during the last three months of the epidemic, the proportionate mortality deduced from the tables published, is nearly equal, *that* for the first month, September, is much smaller. This, M. Louis explains by a source of error we have already adverted to, that many more made their illness known to the public authorities at the commencement of the epidemic than during the subsequent months. The author's general conclusion from these particulars is thus expressed:

"If on account of errors in the official documents, and of the small number of facts collected by the commission, it is not possible to draw strictly exact conclusions either from one or from the other, that the mortality of the disease and the proportion of severe to mild cases were the same at different periods of the epidemic, at least these documents, on a careful study, do not contradict that supposition, but, on the contrary, are favorable to it. The uniformity of the symptoms and lesions observed at different periods of the epidemic seems to give an affirmative answer to the question, Were the character and severity of the yellow fever of Gibraltar of 1828, the same at different periods of the epidemic?" (p. 285.)

It will occur to the reader that he has constantly heard of the rise, height, and decline of epidemics. So far as that of Gibraltar was concerned, the argument of the author tends to show that these words can apply only to the prevalence of the disease—the operation of its cause on a varying number of persons, not to its intensity. That in this sense the words had a correct reference to this epidemic is manifest from the official returns, by which we learn that in September 930 persons were ill, of whom 150 died; in October, 3050, of whom 703 died; in November, 1040, with 251 deaths; and in December, 175, with 47 deaths. This uniformity of malignancy has not, up to the present time, been regarded as characteristic of epidemics; on the contrary, they have been considered as varying in the following manner: At the beginning the proportion of persons attacked is small, but the mortality is great; during the increase and height the number attacked is much greater, but the proportionate mortality is diminished; and during the decline, there is a decrease both of the number attacked and of the fatality.

There is a good chapter on diagnosis. Of course in severe and fatal cases, especially should there be a manifest epidemic, there can be no difficulty. The black vomit, the yellowness of the skin, and the results of autopsies, must render the nature of the disease manifest. In mild cases, however, where there is no declared epidemic, difficulty may arise and the author's directions for surmounting it are of value. He says, in mild cases, all the more or less characteristic symptoms were often wanting. Frequently there were no vomitings of any kind, never black vomit, nor black dejections, nor yellowness, nor anxiety. The disease appeared to consist of some slight febrile symptoms, to which were joined a more or less intense headach, pain in the limbs, back, and loins; sometimes, usually even, redness of the eyes; a weakness so moderate, that many patients did not keep their beds. In cases of this sort, occurring at the commencement of an epidemic, the character of which has not been recognized, and where the patients observed are isolated, it is not only impossible to recognize the disease, but we should scarcely even suspect it. It might be taken for an ephemeral fever, the character of which cannot always be determined, or in cases where the febrile symptoms are accompanied by epigastric pain and nausea, the disease might be considered a slight gastritis; and although, according to observation, the return of strength does not take place in yellow-fever patients after a space of time proportioned to the symptoms and duration of the fever, still so many causes may retard convalescence from the most common diseases, that yellow fever could not be suspected from this consideration alone. But if many similar cases were observed in a short space of time, in the months of August and September, and in the latitude where the yellow fever prevails; if the eyes were injected from the commencement, the countenance red, the headach intense, the epigastrium sensible on pressure, we should strongly suspect this disease, although the existence of an epidemic has not been declared. There would be no doubt as to this point, even if the symptoms existed in the slightest degree only, where the disease attacked all the members, or the greater part of the members of one family, and in a short space of time; since, of diseases of this kind there is no other than yellow fever, which would attack a great number of persons of the same family in so short a space of time.

The very important question: "Does a first attack of yellow fever preserve from a second?"—a question with which another is very directly connected, that of the contagion of this disease, is discussed at considerable length. A commission of thirteen medical men, British, French, and Spanish, was appointed for its examination, and of this commission M. Louis was president.

The question was examined under a double point of view: 1st. If an individual who had had the yellow fever in Europe was susceptible of a second attack of the same disease in Europe. 2d. If one who had had the disease in Europe, could have it a second time in America, and *vice versâ*. Our space does not permit us to describe the precautions taken by the commission to avoid every supposable source of error, or the very accurate detail of circumstances and calculation from which it deduced its conclusion. This conclusion is, that ***second attacks are more rare in the case of yellow fever than of smallpox itself***, that an individual once attacked, even in the slightest degree, is, with very rare exceptions, exempt for ever from future attacks; and this is true, not only where the first attack and second exposure have taken place both in Europe, but where the attack and exposure have been in different continents. M. Louis answers in the valuable corollaries deduced by Dr. Pym from his investigation of the same subject with the same result: 1st. That the persons who have already had yellow fever may remain without risk in a town where it is prevailing epidemically, as did the inhabitants of Gibraltar, and that the care of the sick should, as much as possible, be intrusted to those who have already experienced an attack. To these the commission have added the important conclusion, that the same fact ought to have a great influence in the selection of troops for colonies where yellow fever prevails habitually.

We regard these conclusions respecting the immunity of patients previously affected with yellow fever, as of the highest pathological as well as practical interest: they derive an increase of importance from the analogous conclusions arrived at in regard to the typhus fever of this country, by some of our best recent authorities in Scotland and Ireland.

Two orders of facts, of an entirely different nature, are compared in the chapter on treatment. The practice adopted by the medical staff of the army has no resemblance to that of the private practitioners of Gibraltar. The former employed at the commencement of the epidemic large and repeated general bleedings; but they soon modified this practice, employing subsequently general bleedings only as an auxiliary to purgatives and large doses of calomel; these latter remedies, and, with some practitioners, mercurial frictions, constituting the staple of the treatment. The mortality resulting from the disease under this plan, was one in four and a half. The Spanish physicians, on the other hand, employed bleeding very moderately, and only at the commencement of the disease, moved the bowels by very gentle laxatives, or, in the advanced stage of enemata only, and gave mercury only in a few very severe cases. The mortality under this plan was only one in six, a proportion which led the population of Gibraltar to consider the Spanish physicians as much more successful than the British. But this difference was only apparent, for when we reflect that the mortality among women and children was much less considerable than among men, and when we deduct the number of these last from the general sum, we find the mortality among the civil, male and adult, population, the same as among soldiers, one in four and a half.

In the absence of any plan of cure, of which the value is confirmed by experience, M. Louis suggests that which appears to him to be pointed out by the symptoms as a basis. General bloodletting should be resorted to at the commencement of the disease, and the quantity of blood drawn should be in proportion to the febrile symptoms. He suggests fifteen or sixteen ounces as a suitable amount of blood, and such a bleeding might be repeated within the first twenty-four hours where the patient is very strong and the fever intense.

The effects of the bloodletting should be seconded by the use of cool and slightly acid drinks, such as lemonade, orangeade, or a solution of gum-syrup, if the stomach bears that better than any other kind of drink; and, if possible, the patient should consume from two to three pints, or even more in the course of the twenty-four hours, provided so much liquid does not excite vomiting. The intestinal canal should be evacuated by means of mild enemata, repeated two or three times in the course of twenty-four hours; and emollient fomentations should be applied to the epigastrium.

In reply to his own question, "If the vomitings should suddenly become very frequent, or the epigastric pain very severe, a general blood-letting having been made, would an application of leeches to the epigastric region be advisable?" The author suggests that recourse to such means should depend on the febrile symptoms. Where these are diminished he would abstain from such means: where, however, they are unabated or slightly increased and the patient is strong, he thinks we should be justified in taking eight or ten ounces more of blood from a vein, or in the application of twenty leeches to the epigastrium. He considers the means to be employed for checking hemorrhage, and especially gastro-intestinal hemorrhage; and remarking that such hemorrhage was not less frequent when physicians bled largely, than when they had nearly abandoned bleeding, he does not regard it as a means of preventing such hemorrhage; and finds in the smallness of the pulse, and diminution of temperature, an additional reason for abstaining from it. He recommends that something astringent should be employed, and suggests the intestines as the most suitable organs for its application. We presume he means that the astringent should be used in the form of enemata.

The lesion of the liver does not escape his attention; but being ignorant of the therapeutic agent calculated to combat it, he is obliged to leave to time, chance, and the acuteness of future observers, the discovery of its remedy, experience having proved that no reliance is to be placed on mercurial preparations of any sort.

He disapproves of ether and other energetic antispasmodics, on account of the inflammatory condition of the stomach; but thinks a trial might be made of opiate preparations, which are better adapted to the state of the stomach, and the free employment of which is permitted by the condition of the cerebral organs.

In mild cases, where the febrile symptoms are inconsiderable and the headach moderate, cool drinks and emollient enemata appear to be the only remedies which it is advisable to employ.

We have confined ourselves to describing the remedies suggested by the author, without detailing his reasons for their adoption. All these are founded, partly on the symptoms, but especially on the results of the autopsies, which we have detailed at considerable length. This method of cure deserves the credit of being very *rational;* but we should have felt more confidence in it, and so, too, we are sure, would M. Louis, had it been empirical, and had its author been able to state that by means of it, he had reduced the mortality from one in four and a half to some smaller ratio. We fear there are many *opprobria medicorum,* but of all these, epidemics are the most opprobrious. They seem to go very much their own way in spite of the doctor.

In his concluding chapter, M. Louis examines the question: "Is yellow fever ever sporadic at Gibraltar?" and after a careful analysis of forty-five cases, nineteen of which were fatal, and twenty-six of which recovered, and which occurred at various periods when no epidemic was prevailing, he answers this question in the affirmative. Our limits do not permit us to accompany the author in his analysis of these cases, but we think it amply sufficient to establish the affirmative of the question. In concluding this subject and his volume, he says,

"This conclusion (that yellow fever sometimes exists sporadically at Gibraltar) being admitted as correct, the question of the mode in which yellow fever is communicated would not be settled, its non-contagiousness would not follow. For we see every day the most incontestably contagious diseases, as, for example, the smallpox, under sporadic forms, and appearing only at distant intervals of time. All, then, that we can conclude, the existence of sporadic cases of yellow fever being admitted, is, that this fever may be developed at Gibraltar, independently of anything coming from America. To conclude absolutely that the yellow fever is not contagious, would be to go beyond our facts, and if this conclusion be the expression of the truth, it can result only from another class of facts." (p. 374.)

We take leave of this volume, but not finally, we trust, of its author, with our previously high opinion of his powers as an observer and a reasoner, exalted by its perusal. The fruit of his labours on yellow fever is certainly rather in prospect than possession; but he has given a precision to the whole subject, of which it was previously destitute, and this is the only mode by which we can be invested with a power over the disease, of which we are at present unhappily destitute. The language of the translation is very creditable to Dr. Shattuck; and we earnestly recommend his book to the attention of all our readers who are resident in, or likely to be called to visit the haunts of the fever of which it treats.

---

